# Cooperative Effect of Ammonium Polyphosphate and Talcum for Enhancing Fire-Proofing Performance of Silicone Rubber-Based Insulators via Formation of a HIGH-Strength Barrier Layer

**DOI:** 10.3390/polym18020283

**Published:** 2026-01-20

**Authors:** Dong Zhao, Yihan Jiang, Yong Fang, Tingwei Wang, Yucai Shen

**Affiliations:** 1 School of Materials Engineering, Changzhou Vocational Institute of Industry Technology, Changzhou 213164, China; zhaodong@ciit.edu.cn (D.Z.); 17706129938@163.com (Y.J.); 2 College of Material Science and Engineering, Nanjing Tech University, Nanjing 211816, China; 202361203290@njtech.edu.cn (Y.F.); wangtw@njtech.edu.cn (T.W.)

**Keywords:** silicone rubber, composites, flame retardancy, high temperature resistance, high-strength char layer

## Abstract

Enhancing the flame retardancy of polymeric materials by adding only eco-friendly ammonium polyphosphate (APP) while simultaneously maintaining high-temperature resistance has become a challenge. Talcum has been introduced as a cooperative agent into the silicone rubber/APP system to investigate the effect of talcum on flame retardancy, thermal stability, and high-temperature resistance. The machining process induces the orientation of talcum in the system. The ceramifiable silicone rubber blends containing oriented talcum (e.g., sample SA_6_T_4_) exhibited superb flame retardancy, including an LOI of 29.4%, a UL-94 rating of V-0, and a peak heat release rate (PHRR) of 250.2 kW·m^−2^. More importantly, the blends present excellent thermal stability and high-temperature resistance, characterized by outstanding self-supporting properties and dimensional stability. Based on the structural analysis of the blends and their residues, the made of action for the improved flame retardancy may be attributed to the formation of a compact barrier layer. This layer is formed by oriented talcum platelets combined with phosphoric acid, from the thermal decomposition of APP, promoting crosslinking, thereby achieving a good inhibition barrier to inhibit heat feedback from the condensation zone. The excellent thermal stability and high-temperature resistance of the ceramifiable silicone rubber blends may be ascribed to a cooperative effect between APP and talcum at high temperatures, which facilitates the formation of ceramic structures. The novel ceramifiable silicone rubber composite has potential applications as flame-retardant sealing components for rail transit equipment and encapsulation materials for new energy battery modules.

## 1. Introduction

Silicone rubber with a unique structure exhibits outstanding electrical insulation properties, high thermal stability, and high-temperature resistance [1,2,3]. Therefore, it has been widely used in rail transit equipment, new energy battery encapsulation, the wire/cable industry, the nuclear industry, and in aerospace applications [4,5,6,7]. In contrast to organic polyolefins, silicone rubber exposed to open fire or high temperatures can be converted into inorganic silica residues. Hence, silicone rubber has been utilized to prepare ceramifiable polymer formulations for high-temperature-resistant cable insulation [8,9]. This type of material can be converted into ceramic residues of high temperatures, which can protect copper wires from melting. Based on this characteristic, this ceramifiable silicone rubber composite has also attracted intensive research attention as a flame-retardant encapsulation material for power batteries. Facing fires, the more complete and compact the ceramic residue is, the better its thermal protection performance. The completeness and compactness of the ceramic residue depend on many factors, such as the degradation behavior of the polymer matrix, filler type, and pyrolysis conditions [10,11,12,13,14]. Unfortunately, silicone rubber is relatively flammable and will burn continuously once ignited [4], which significantly disrupts the ceramization process of ceramifiable silicone rubber compositions. Therefore, it is necessary to reduce the combustibility of silicone rubber and develop flame-retardant silicone rubber blends.

To achieve acceptable flame retardant efficiency in polymeric compositions, the combination of APP with other agents, such as inorganic fillers, has been utilized [15,16,17,18,19,20]. Modified montmorillonite intercalation cobalt compounds (Co-Mt) display a cooperative effect with APP when used in polypropylene. A cooperative effect of APP and aliphatic polyamines on the flame retardant properties of epoxy composites has been noted [16]. Blends of epoxy resin with amine-modified APP display better flame retardant performance compared to that of pure APP. Addition of ammonium polyphosphate and zinc borate into an EVA/MP/OMMT system improves flame retardancy and ceramization [17]. The incorporation of APP and CaCO_3_ as catalytic agents can remarkably enhance the thermal stability and flame retardancy of silicone rubber blends [4]. This may be attributed to significantly improved quality of the condensed-phase char layers and ceramic layers deposited on the surface of the degrading polymer matrix. The effects of APP, ADP, and OPS on the thermal and ablative properties of RTV silicone rubber have been evaluated [18]. APP or ADP combined with OPS exhibit a significant cooperative effect in improving the flame retardancy and high-temperature resistance of silicone rubber blends, due to the enhanced quality of condensed phase action.

The flame retardant effect of APP is based on the dilution effect and the protective action of the P-O-enriched char layer against combustibles and heat transfer between the gaseous flame zone and the pyrolyzing melt zone [21]. The formation of the char layer represents condensed-phase flame retardant action. It is generally accepted that flame retardant efficiency is improved when the barrier action of the char layer is enhanced. Talcum, a hydrated silicate with a lamellar structure, can significantly improve the thermal and barrier properties of polymer matrices [22]. Because of the special structure of talcum, oriented talcum and APP can cooperatively enhance the strength of the char layer and contribute to the improvement of flame retardancy. The chemical component of talcum—including oxides and hydroxides of magnesium—exists as a sandwich structure between two silica layers [22]. The thermal decomposition products of APP may react with alkaline-earth metal ions at high temperatures [23,24]. Therefore, ceramization might occur between APP and talcum at high temperatures. Owing to the unique structure of talcum, talcum and the decomposition products of APP can form a specially enhanced ash layer, which further improves flame retardancy and high-temperature resistance. The improved flame retardancy and enhanced high-temperature resistance of filled silicone rubber ensure the integrity of wires and cables, thereby guaranteeing the normal transmission of electrical power and signals during fire accidents.

The preparation of silicone rubber/talcum/APP blends with excellent flame retardancy, thermal stability, and high-temperature resistance has been achieved. To improve the flame retardant efficiency of APP, talcum was introduced into the silicone rubber matrix as a cooperative agent. The effect of the APP/talcum mass ratio on the flame retardancy and high-temperature resistance of the silicone rubber compositions was investigated using the limited oxygen index (LOI), vertical burning test (UL-94), cone calorimeter test (CC), and ceramification property tests. Moreover, cooperative flame retardant action was evaluated using thermogravimetric analysis (TGA) and microstructure evaluation of the silicone rubber/talcum/APP structures. In addition, the high-temperature resistance of the filled silicone rubber compositions was evaluated through tests of self-supporting properties, flexural strength, and linear shrinkage. A possible ceramization process between APP and talcum at high temperatures has been demonstrated.

## 2. Materials and Methods

### 2.1. Materials

**Figure A1:**
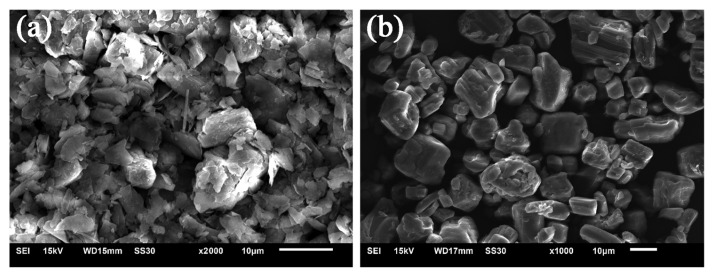
SEM images of talcum (**a**) and APP (**b**).

A high-temperature vulcanized silicone rubber (SR) was obtained from Jiangsu Tianchen New Material Company (771-u; Jiangsu Tianchen New Material Company, Yangzhou, China). Bis(tert-butyldioxyisopropyl) benzene (BIPB), used as the curing agent, was purchased from Hunan Yixiang Technology Company (FARIDA BIPB 96; Hunan Yixiang Technology Company, Changsha, China). Ammonium polyphosphate (APP) was supplied by Jiangsu Xingxing Fire-retardants Co., Ltd. (APP 1000; particle size D50 ≤ 20 μm; Zhenjiang, China). Talcum with a lamellar structure was sourced from Qingdao Lukuang Talcum Powder Co., Ltd. (average particle size: 10 μm; Shandong, China). The microstructures of talcum and APP are shown in Figure A1, and the elemental compositions of talcum are presented in Table A1.

### 2.2. Preparation of Ceramifiable Silicone Rubber Composite with Orientated Talcum

**Figure 1:**
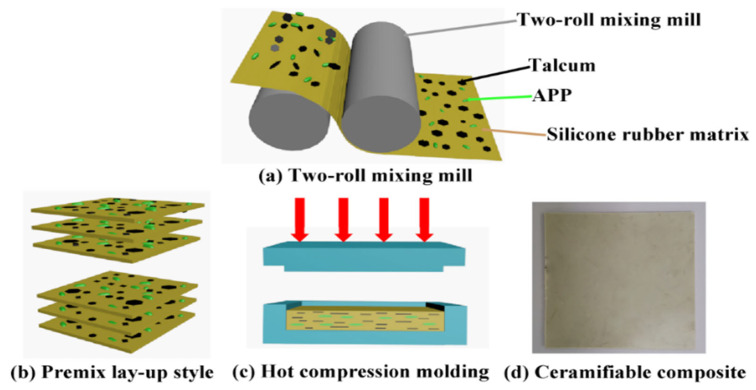
Schematic illustration for the preparation process of ceramifiable silicone rubber composite.

APP and talcum were dried in a vacuum oven at 80 °C for 10 h. Mixed fillers (APP and talcum) were prepared using a high-speed mixer (Zhangjiagang Yunfan Machinery Co., Ltd., Suzhou, China) for 30 min. All silicone rubber composites were mixed with a two-roll mixing mill (Shiyan Precision Instruments Co., Ltd., Dongguan, China). The silicone rubber matrix was first softened at room temperature, and then the mixed fillers (APP and talcum) were added, followed by mixing for approximately 15 min. BIPB was added last until a homogeneous mixture was obtained. The distance between the two rolls was adjusted to approximately 0.2 mm. The premix was removed from the rolls using a putty knife. The formulations of the ceramifiable silicone rubber composites used in this study are presented in Table 1. All tested samples were fabricated into flat sheets via hot pressing at 180 °C for 10 min under a pressure of 10 MPa. The preparation process is illustrated in Figure 1. Based on the well-established conclusion that filler orientation contributes to the optimization of material properties, this study focuses on the direct verification of the oriented structure of talcum powder, thus resulting in the limitation of not setting up a non-oriented talc control group. Future research will further verify the independent effect of talc orientation by preparing non-oriented samples via the low-shear mixing process and direct hot-pressing process.

### 2.3. Characterization

The elemental compositions of talcum were determined via the X-ray fluorescence (XRF) method using an ADVANT’XP XRF spectrometer (Antonpa (Shanghai) Trading Co., Ltd., Shanghai, China). All silicone rubber composites were fired in a muffle furnace (Hefei Kejing Materials Technology Co., Ltd., Hefei, China) from 25 °C to different target temperatures (600, 800, 900, and 1000 °C) at a heating rate of 10 °C/min and held at the maximum temperature for 30 min.

The self-supporting property was evaluated as follows: First, a sample with dimensions of 50 × 5 × 2 mm^3^ was placed on two parallel refractory bricks, with its long axis perpendicular to the edges of the bricks. The distance between the two bricks was approximately 12 mm. The sample was then heated from room temperature to the target temperature at a rate of 10 °C/min in a muffle furnace and held for 30 min, after which the bending angle of the sample between the bricks was recorded.

The electrical insulation properties of composite sheets (100 × 100 × 2 mm^3^) were measured using a high-resistance meter (EST120; Beijing Zhongxi Huada Technology Co., Ltd., Beijing, China) following the IEC 60093:1980 standard.

The microstructures of the composites and their ceramic residues were characterized by scanning electron microscopy (SEM; JSM-6300, JEOL, Tokyo, Japan) and energy-dispersive X-ray spectroscopy (EDX; Thermo Scientific, Waltham, MA, USA). The limiting oxygen index (LOI) was tested using an oxygen index meter (HC-2; Shanghai Le Ao Test Instrument Co., Ltd., Shanghai, China) in accordance with GB/T 2406:2008. The UL-94 vertical burning rating was determined using a vertical burning tester (ZF-621; Qingdao Zhongbang Instrument Co., Ltd., Qingdao, China) following ASTM D3801:2020 (UL-94) standards. Cone calorimeter (CC) tests were conducted on a cone calorimeter (FTT, Sussex, UK) at a heat flux of 50 kW·m^−2^ in accordance with ISO 5660:2015.

Volatiles released during thermal degradation were analyzed in situ via thermogravimetry-Fourier transform infrared spectroscopy (TG-FTIR), coupling a thermogravimetric analyzer (Q20; TA Instruments, New Castle, DE, USA) and a Fourier-transform infrared (FTIR) spectrometer (Nicolet iS 10; Thermo Fisher Scientific, Waltham, MA, USA). Tests were performed under a nitrogen atmosphere from 20 to 800 °C at a heating rate of 10 °C/min.

Thermal stability was evaluated using a thermogravimetric analyzer (TGA-601; Nanjing Huicheng Instrument Co., Ltd., Nanjing, China) with sample masses of 5–10 mg. Tests were conducted in air from 50 to 800 °C at a heating rate of 10 °C/min. X-ray diffraction (XRD) analysis was performed on a diffractometer (Rigaku Corporation, Tokyo, Japan) with Cu Kα radiation, over a 2θ range of 10–70° at a scanning rate of 10 °/min.

FTIR analysis was carried out using an FTIR spectrometer (IR-2000; Tianjin Jingtuo Instrument Technology Co., Ltd., Tianjin, China) over a wavenumber range of 4000–400 cm^−1^ at a resolution of 4 cm^−1^. The flexural strength of fired samples was measured using a universal testing machine (WDW-20; Wenteng Test Instruments, Jinan, China) via the three-point bending method in accordance with ISO 14704:2016 at a crosshead speed of 0.5 mm/min. All results represent the average of five replicate tests.

The linear shrinkage (L) of the composites was calculated using Equation (1):

(1) $$L=\frac{L_{t}-L_{0}}{L_{0}}\times 100\%$$

where *L_0_* (mm) is the length of the sample before firing, and L_t_ (mm) is the length of the sample after firing at high temperatures.

The bulk density of the ceramic residues was determined via the Archimedes method with deionized water as the immersion liquid, following ASTM C373-88:2006 standards.

## 3. Results

### 3.1. Morphologies of the Ceramifiable Silicone Rubber Composites

**Figure 2:**
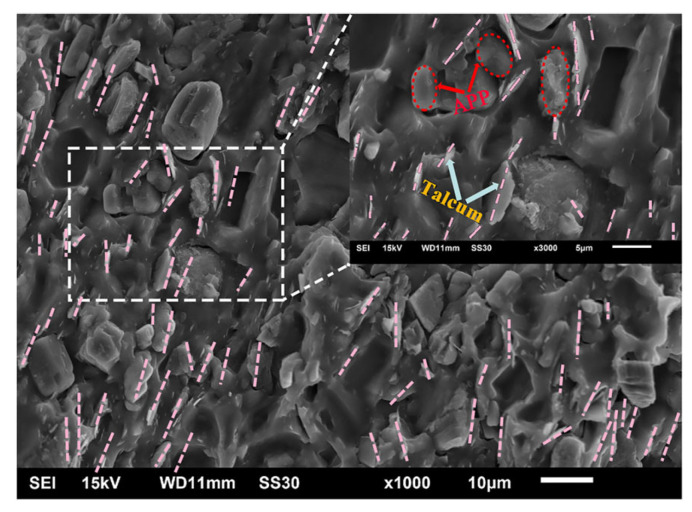
SEM image of sample SA_6_T_4_ cross-section before ceramification, taken under magnification of 1000× and 3000×.

**Figure A2:**
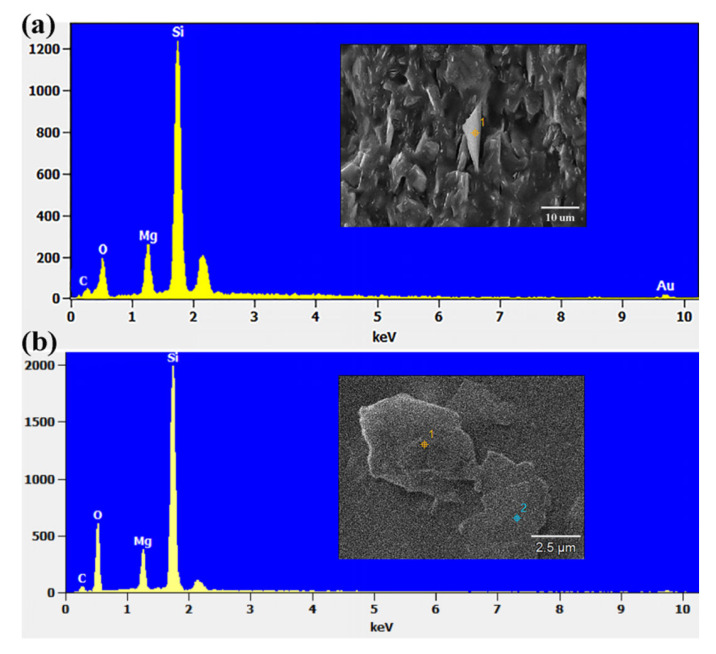
EDX spectra of internal (**a**) and surface (**b**) domain of sample SA_6_T_4_. Note: The numbers “1” or “2” in the SEM images denote the scanning positions for EDX measurements.

The SEM image of sample SA_6_T_4_ is shown in Figure 2, and the SEM image of layered talcum is presented in Figure A1. It can be seen that there are some agglomerations in the talcum powder. However, no obvious agglomerated talcum is observed in the ceramifiable silicone rubber composites prepared via two-roll milling (Figure 2). Owing to the shear force of the two-roll mixing mill, excellent dispersion of layered talcum in the silicone rubber matrix can be achieved, and the talcum’s structure is transformed from laminated to single-layered [25,26]. Figure 2 and Figure A2 show that APP particles and single-layered talcum are uniformly dispersed in the silicone rubber matrix, and the oriented arrangement of talcum platelets in the silicone rubber matrix can be clearly observed, which confirms that the strong shearing and hot-pressing effects during the processing can induce the effective orientation of talcum platelets in the composite [27]. In the oriented silicone rubber/talcum/APP composites, some talcum platelets appear as 1D rods (indicated by pink dotted lines) and some APP particles appear as 3D blocks (indicated by red circles) in Figure 2. Furthermore, oriented talcum can also be observed in the surface region of sample SA_6_T_4_ (Figure 10b and Figure A2b) and sample SA_8_T_2_ (Figure 10a). These SEM results demonstrate that the actual microstructures of the ceramifiable silicone rubber composites are consistent with the expected design.

### 3.2. Electrical Insulation Property

Table 2 shows that sample Sr exhibits higher volume resistivity and surface resistivity than those of the ceramifiable silicone rubber composites. The reduction in electrical insulation of the ceramifiable composites can be attributed to free ammonium ions released from APP. Therefore, it can also be observed that the volume resistivity and surface resistivity of the ceramifiable silicone rubber composites decrease with an increasing APP/talcum ratio. Although Table A1 indicates that talcum contains a large amount of magnesium, the oxides and hydroxides of magnesium exist as a sandwich structure between two silica layers. This endows each talcum lamella with a certain degree of electrical insulation [22,28].

### 3.3. Flame Retardancy of Ceramifiable Silicone Rubber Composites

The limiting oxygen index (LOI) and UL-94 vertical burning tests were used to evaluate the flame-retardant properties of the ceramifiable silicone rubber composites. The results of the LOI and UL-94 tests are summarized in Table 3. The LOI value of pure silicone rubber (Sr) is 20.5%, and it has no UL-94 rating. With the incorporation of APP and talcum, the LOI values and UL-94 ratings of the ceramifiable composites are significantly improved. When the mass ratio of APP to talcum is 2:8, the LOI value of sample SA_2_T_8_ is 24.5%. With a further increase in the mass ratio of APP to talcum, the UL-94 rating reaches V-0, and the LOI value of sample SA_8_T_2_ reaches up to 29.9%.

**Figure 3:**
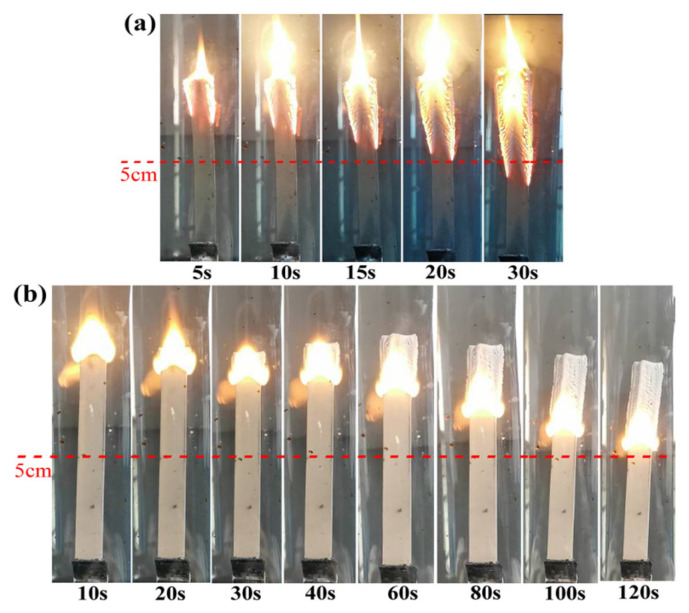
Digital photos of Sr (**a**) and sample SA_6_T_4_ (**b**) under the LOI of 30.0%.

The digital photos (Figure 3) depict the burning processes of samples Sr and SA_6_T_4_ at an LOI of 30.0%. For sample Sr, the burning was extremely intense, and the flame reached the 5 cm mark in nearly 30 s. When APP and talcum were incorporated into the silicone rubber matrix, the flame retardancy of the composite was significantly enhanced. For sample SA_6_T_4_, the flame did not reach the 5 cm mark until approximately 120 s, indicating that the addition of APP and talcum can remarkably reduce the flame spread rate of the ceramifiable silicone rubber composites [21,29]. Compared with the ash residue of sample Sr, the ash residue of sample SA_6_T_4_ is more compact.

**Figure A3:**
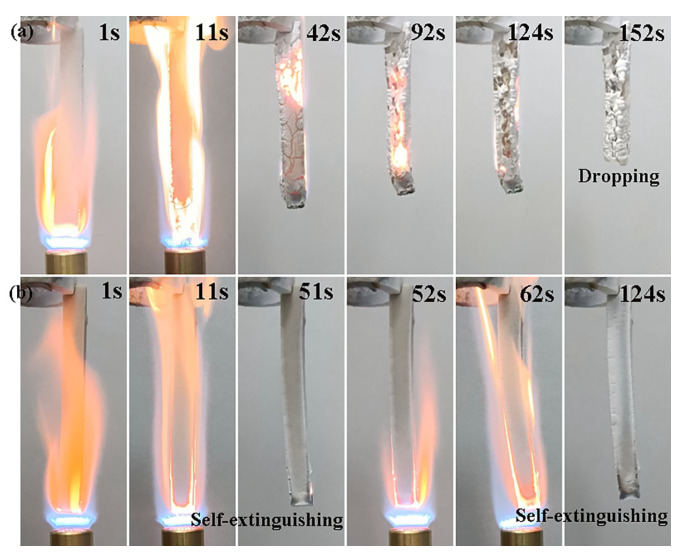
Vertical combustion process of samples Sr (**a**) and SA_6_T_4_ (**b**).

The vertical burning processes of samples Sr and SA_6_T_4_ were recorded in detail, as shown in Figure A3. After being continuously ignited for 10 s, sample Sr underwent rapid flame propagation and sustained combustion owing to the presence of abundant flammable groups in the silicone rubber molecular chains. Eventually, it was completely burned, accompanied by severe dropping and structural damage. The vertical burning performance of sample SA_2_T_8_ was not significantly improved, exhibiting a similar combustion behavior to that of sample Sr. A remarkable transition in vertical burning behavior was observed for sample SA_6_T_4_. Specifically, the sample self-extinguished after 40 s and 62 s following the first and second 10 s continuous ignition, respectively. Notably, sample SA_6_T_4_ maintained its intact morphology throughout the vertical burning process, without any occurrence of dropping or obvious structural damage. Samples SA_8_T_2_ and SA_6_T_4_ exhibited similar vertical burning test results.

**Figure 4:**
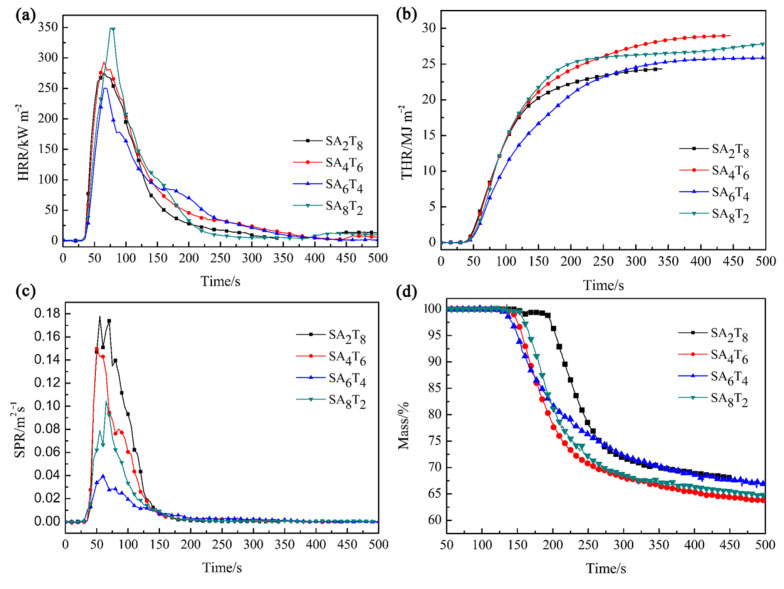
HRR (**a**), THR (**b**), SPR (**c**), and mass loss (**d**) curves of all silicone rubber composites at flux of 50 kW·m^−2^.

The effect of APP and talcum on the combustion behaviors of ceramifiable silicone rubber composites was evaluated via cone calorimetry tests. The heat release rate (HRR), total heat release (THR), smoke production rate (SPR), and mass loss rate (MLR) curves of the ceramifiable silicone rubber composites are shown in Figure 4, and the corresponding characteristic parameters are summarized in Table 4. It is clear that the ceramifiable silicone rubber composites with different mass ratios of APP to talcum exhibit distinct combustion behaviors. The peak heat release rate (PHRR) values of samples SA_2_T_8_, SA_4_T_6_, SA_6_T_4_, and SA_8_T_2_ are 275.3, 293.3, 250.2, and 348.6 kW·m^−2^, respectively. The THR values are 24.3, 28.9, 25.9, and 27.8 MJ·m^−2^, respectively. The peak smoke production rate (PSPR) values are 1.8 × 10^−1^, 1.5 × 10^−1^, 0.4 × 10^−1^, and 1.0 × 10^−1^ m^2^·s^−1^, respectively. The residual yield values of the composites after cone calorimetry tests are 67.8, 62.3, 65.5, and 62.4%, respectively.

**Figure 5:**
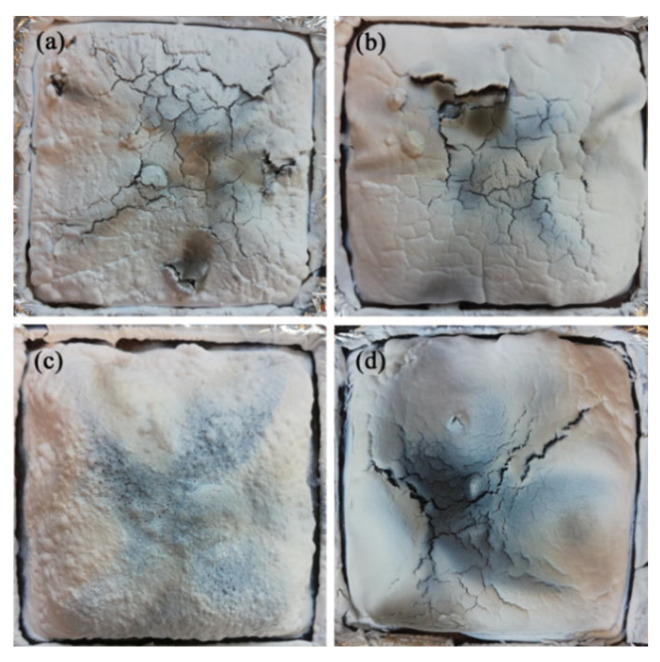
Digital photographs of the residues at a flux of 50 kW·m^−2^. (**a**) SA_2_T_8_; (**b**) SA_4_T_6_; (**c**) SA_6_T_4_; (**d**) SA_8_T_2_.

The digital photographs of the residues from samples SA_2_T_8_, SA_4_T_6_, SA_6_T_4_, and SA_8_T_2_ after the cone calorimetry test are shown in Figure 5. The residue of SA_6_T_4_ is noticeably more compact than those of SA_2_T_8_, SA_4_T_6_, and SA_8_T_2_, with no visible cracks. Similar results are also observed in the microstructural analysis (Figure 10). The formation of cracks is attributed to the rapid release of gases [12]. However, the talcum lamellae act as a physical barrier that delays and inhibits the diffusion of combustible gases and other volatiles (e.g., steam and ammonia) into the air. APP forms phosphoric acid via thermal decomposition, which adheres to the silicone rubber matrix and talcum, thereby suppressing the transfer of heat and combustible gases. Moreover, the ceramic residue formed by the interaction between phosphoric acid and talcum further serves as a physical barrier, inhibiting the release of smoke, gases, and heat transfer. Thus, the key to enhancing the flame retardancy of ceramifiable silicone rubber composites lies in the mass ratio of APP to talcum. For sample SA_2_T_8_, there is too little APP to generate sufficient phosphoric acid. For sample SA_8_T_2_, a large amount of gas (ammonia and steam) is generated from the excess APP. The rapid release of large quantities of gas can impair the compactness of the barrier layer. Therefore, either too much or too little APP fails to effectively improve the flame retardancy of the ceramifiable silicone rubber composites.

**Figure 6:**
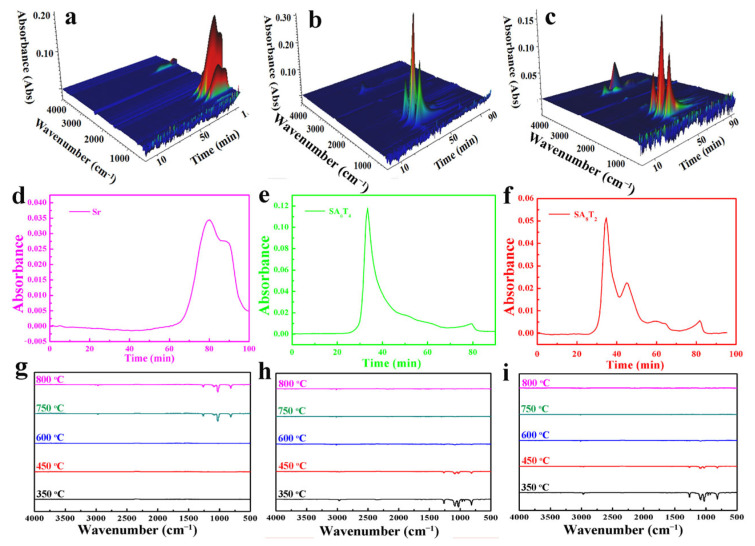
Three-dimensional TG-FTIR spectra of pyrolysis products for Sr (**a**), SA_6_T_4_ (**b**) and SA_8_T_2_ (**c**); absorbance intensity changes of total pyrolysis products for Sr (**d**), SA_6_T_4_ (**e**) and SA_8_T_2_ (**f**); and FT-IR spectra of pyrolysis products for Sr (**g**), SA_6_T_4_ (**h**) and SA_8_T_2_ (**i**) at different times during thermal degradation.

The thermal degradation behaviors of samples SA_6_T_4_ and SA_8_T_2_ under a nitrogen atmosphere were analyzed via Thermogravimetry-Fourier transform infrared spectroscopy (TG-FTIR) and compared with that of pure silicone rubber (Sr). The FTIR spectra of the evolved gases from SA_6_T_4_, SA_8_T_2_, and Sr are distinct, indicating different thermal degradation processes (Figure 6a–f). For Sr, characteristic peaks of the Si–O–Si group at 1082 and 1027 cm^−1^, and the Si–CH_3_ group at 1259 and 812 cm^−1^, appeared in the temperature range of ~400–800 °C (Figure 6g) [30,31]. However, these peaks did not appear for samples SA_6_T_4_ and SA_8_T_2_ within the same temperature range (Figure 6h,i). This can be attributed to the restriction of silicon-based small molecules by laminar talcum attached with phosphoric acid. For samples SA_6_T_4_ and SA_8_T_2_, characteristic peaks of NH_3_ at 964 cm^−1^ were observed in the range of ~300–450 °C [32] (Figure 6h,i). The release of NH_3_ was ascribed to the thermal decomposition of APP. It is also worth noting that more volatiles were released in the higher temperature range with increasing APP content (Figure 6b,c). These volatiles could impair the compactness of the barrier layer formed in the early stage, thereby adversely affecting the flame retardancy of the composites.

**Figure 7:**
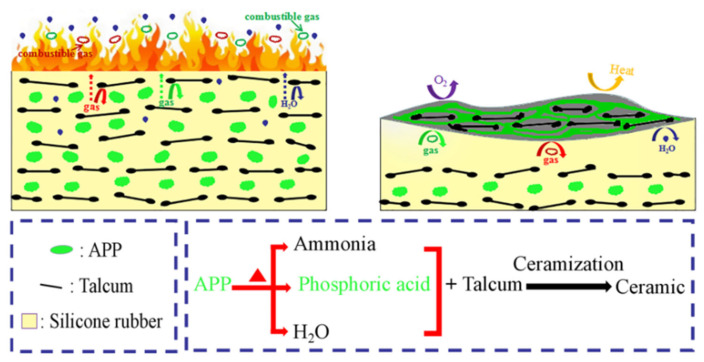
Schematic ceramization mechanisms of the ceramifiable silicone rubber composite.

The ceramization mechanisms of ceramifiable silicone rubber composites are illustrated in Figure 7, based on the results of cone calorimetry tests, thermal analysis, XRD analysis, and TG-FTIR analysis. The decomposition temperature of APP is well-matched with that of the silicone rubber matrix, leading to the generation of phosphoric acid, steam, ammonia, and other volatiles. The generation of ammonia and steam can dilute the surrounding air [33]. Phosphoric acid can adhere to the talcum and silicone rubber matrix, suppressing the transfer of O_2_, combustible gases, and heat. Importantly, phosphoric acid can react with talcum to form a compact and rigid ceramic structural phase. This ceramic structural phase acts as a strengthened physical barrier, insulating the unburned silicone rubber matrix from heat and gases [10].

### 3.4. Self-Supporting Property and Surface Morphology of Ceramifiable Silicone Rubber Composites at High Temperatures

**Figure 8:**
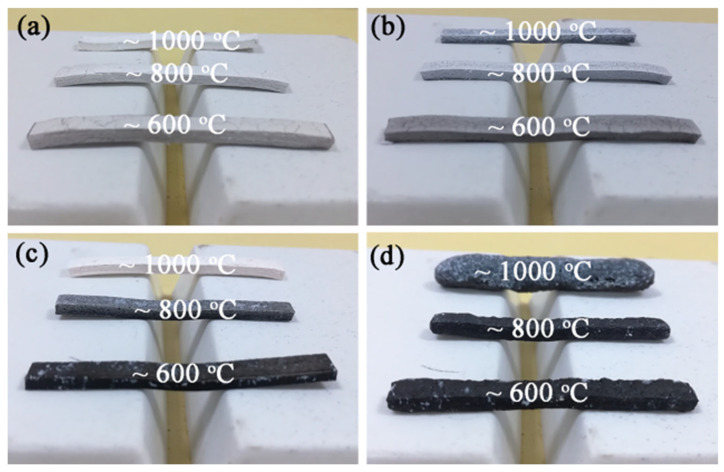
Digital photos of self-supporting of all samples: (**a**) SA_2_T_8_; (**b**) SA_4_T_6_; (**c**) SA_6_T_4_; (**d**) SA_8_T_2_.

**Figure A4:**
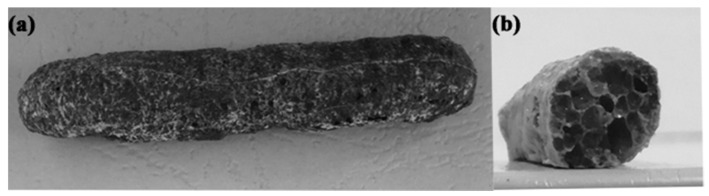
The surface (**a**) and cross-sectional (**b**) morphology of sample SA_8_T_2_ sintering from room temperature to 1000 °C.

The self-supporting property and surface morphology of ceramifiable silicone rubber composites fired from room temperature to high temperatures are illustrated in Figure 8. As the temperature increases from room temperature to various target temperatures (600, 800, and 1000 °C), the self-supporting property of the composites improve, enabling them to support their own weight. The bending angle values of all samples at high temperatures are less than 5°. Ceramifiable silicone rubber composites exhibit excellent self-supporting properties across the temperature range from room temperature to high temperatures, attributed to the presence of talcum adhered with phosphoric acid at low-to-medium temperatures and the formation of crystalline phases via reactions between talcum and phosphoric acid at high temperatures [11,24]. Additionally, an increase in the APP/talcum mass ratio causes the color of the fired specimens to change from white to black. This is primarily due to the presence of phosphoric acid (generated from APP decomposition), which promotes the carbonization of the silicone rubber matrix. The surface morphology of samples SA_2_T_8_, SA_4_T_6_, and SA_6_T_4_ remains smooth and intact at 600, 800, and 1000 °C. However, the surface morphology of sample SA_8_T_2_ fired from room temperature to 600, 800, and 1000 °C becomes irregular, with obvious expansion observed as the temperature increases. The loose and porous structure formed in sample SA_8_T_2_ after firing at 1000 °C is attributed to the release of ammonia and steam from the pyrolysis of excess APP (Figure A4).

### 3.5. Flexural Strength, Linear Shrinkage, and Bulk Density

The flexural strength, linear shrinkage, and bulk density of the fired specimens are presented in Table 5. By comparing the flexural strength of all samples fired at 900 °C, sample SA_6_T_4_ exhibits the highest flexural strength, along with the largest linear shrinkage and highest bulk density. Several factors influence the linear shrinkage of the samples at high temperatures, including gas release, filler melting, and crystalline reactions. Theoretically, linear shrinkage can be reduced with an increase in APP content. Moreover, lamellar talcum can inhibit sample shrinkage due to its poor fluidity and excellent barrier properties. However, the melting of talcum or crystalline reactions between APP and talcum at high temperatures can promote sample shrinkage. For samples SA_2_T_8_, SA_4_T_6_, and SA_6_T_4_, linear shrinkage decreases with an increase in the APP/talcum mass ratio. This is mainly attributed to the melting of talcum, where crystalline reactions between APP and talcum play a dominant role in sample shrinkage. When the APP/talcum mass ratio reaches 8:2, gas release from APP becomes the dominant factor contributing to sample expansion. Additionally, the density trends of all samples fired at 900 °C are consistent with the aforementioned analysis of dimensional changes.

**Figure 9:**
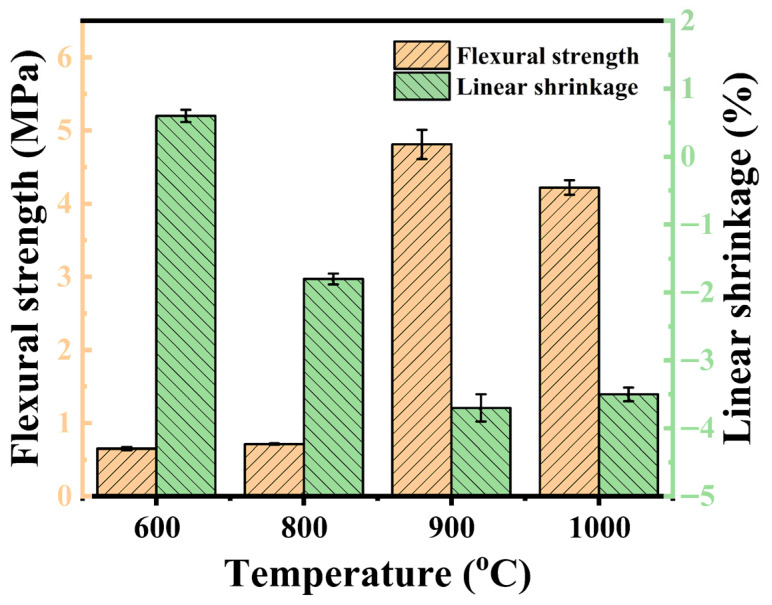
Flexural strength and linear shrinkage of ceramic residues of sample SA_6_T_4_ fired at different temperatures.

Figure 9 shows the flexural strength and linear shrinkage of the ceramic residues of sample SA_6_T_4_ fired at different temperatures. The linear shrinkage of the ceramic residue of sample SA_6_T_4_ fired at 600 and 800 °C is 0.6% and −1.8%, respectively. As the firing temperature further increased to 900 and 1000 °C, the linear shrinkage of the specimens increased to −3.7 and −3.5%. As shown in Figure 13a, talcum cannot melt completely below 800 °C, which is the main reason for the relatively low linear shrinkage of the specimens fired at 600 and 800 °C. Above 900 °C, talcum melts and reacts with other components, resulting in significant shrinkage of sample SA_6_T_4_. The linear shrinkage of the specimen fired at 1000 °C slightly increased compared to that of the specimen fired at 900 °C, likely attributed to the more rapid thermal decomposition of sample SA_6_T_4_ at 1000 °C [34]. Additionally, the flexural strength of the specimens increased from 0.65 to 4.81 MPa as the firing temperature increased from 600 to 900 °C. However, the flexural strength of the specimen fired at 1000 °C slightly decreased compared to that of the specimen fired at 900 °C.

### 3.6. SEM Analysis

**Figure 10:**
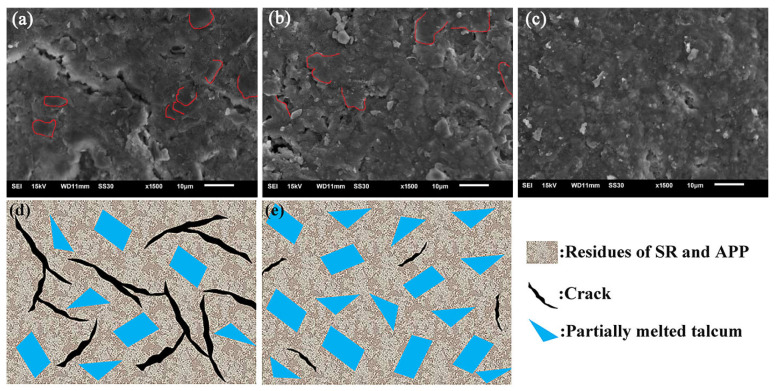
Surface SEM images of sample SA_8_T_2_ after firing from room temperature to 600 °C (**a**); sample SA_6_T_4_ after firing from room temperature to 600 and 900 °C (**b**,**c**). Schematic of the formation of the reinforced physical barrier (**d**,**e**).

The microstructures of the residues from sample SA_8_T_2_ fired from room temperature to 600 °C at a heating rate of 10 °C/min are shown in Figure 10a. Numerous cracks can be observed on the surface of the residue (Figure 10a). In contrast, sample SA_6_T_4_ exhibits a more continuous and compact structure under the same firing conditions (Figure 10b). Moreover, partially melted talcum (indicated by red dotted lines) is visible in both Figure 10a,b. This is because the melting point of talcum is approximately 900 °C; for sample SA_6_T_4_, with an increase in temperature, the presence of phosphoric acid promotes the melting of talcum. The liquid phase derived from talcum acts as a binder to enhance the density of the residue. As shown in Figure 10c, a continuous and dense structure is formed, which results in an increase in bulk density and an improvement in the flexural strength of the fired specimens. Furthermore, this also further verifies the accuracy of the flame-retardant mechanism proposed earlier. Compared to sample SA_8_T_2_, sample SA_6_T_4_ can form a more effective reinforced physical barrier to enhance flame retardancy. The schematic of the reinforced physical barrier formation is illustrated in Figure 10d,e.

### 3.7. Thermogravimetric Analysis (TGA)

**Figure 11:**
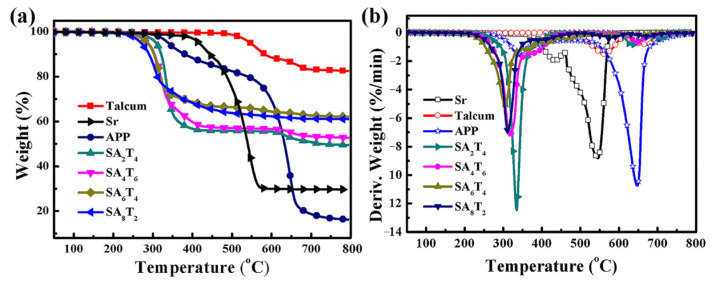
TG (**a**) and DTG (**b**) curves of Sr, talcum, APP, and silicone rubber/ammonium polyphosphate/talcum composites.

**Figure 12:**
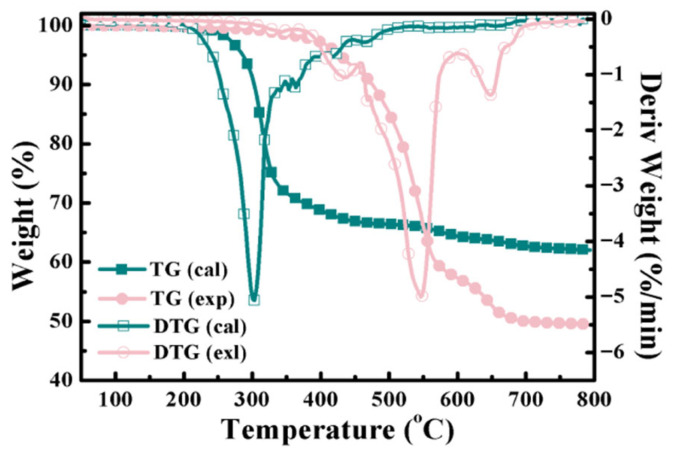
Experimental and calculated TG and DTG curves of sample SA_6_T_4_.

The thermogravimetric (TG) and derivative thermogravimetric (DTG) curves of Sr, APP, talcum, and all the silicone rubber/APP/talcum composites are shown in Figure 11. Synergistic interactions among APP, talcum, and Sr can be revealed by comparing the composites’ experimental and calculated TG and DTG curves. The experimental and calculated TG and DTG curves of sample SA_6_T_4_ are presented in Figure 12.

It can be observed that APP undergoes two stages of thermal decomposition. The first stage occurs at 292.45–403.85 °C, attributed to the elimination of ammonia and water to generate polyphosphoric acid; the second stage takes place at 579.40–694.74 °C, resulting in the volatilization of P–O-containing compounds [23]. The thermal decomposition of Sr starts with an initial stage (383.18–450.71 °C), involving the oxidative decomposition of side chains and the generation of cyclosiloxanes via unzipping reactions. In the second decomposition stage (463.24–565.77 °C), main chain cleavage occurs, producing silica [35]. For talcum, two distinct thermal decomposition stages are observed: the first (490.61–595.71 °C) corresponds to the removal of interlayer water, and the second (638.25–723.32 °C) involves the dehydroxylation of talcum, leading to lattice damage and defects [36]. The total weight loss of talcum is 18.2%, which is consistent with the XRF analysis results.

As presented in Figure 11b, all silicone rubber/APP/talcum composites exhibit a single main thermal decomposition stage. Based on TG analysis results, this stage involves both the decomposition of APP and the degradation of the silicone rubber matrix. Significant differences in this decomposition stage are observed among the composites: the initial decomposition temperature of the composites decreases with an increase in the APP/talcum mass ratio. The initial decomposition temperatures of SA_6_T_4_ and SA_8_T_2_ are similar, which can be attributed to the decomposition of APP or the presence of impurity metal ions in talcum, both of which accelerate the thermal decomposition of the silicone rubber matrix. However, compared with SA_2_T_8_, SA_4_T_6_, and SA_8_T_2_, SA_6_T_4_ exhibits the lowest maximum decomposition rate during this stage. This is ascribed to the interaction between phosphoric acid and talcum (at an APP/talcum mass ratio of 6:4), which forms a barrier layer that effectively inhibits the volatilization of combustible and non-combustible volatiles. Additionally, a weak thermal degradation peak appears above 550 °C for samples SA_2_T_8_, SA_4_T_6_, and SA_8_T_2_, indicating damage to the barrier layer. Furthermore, the residual yield of SA_6_T_4_ at 800 °C is 62.05%, which is higher than those of SA_2_T_8_ (49.55%), SA_4_T_6_ (52.75%), and SA_8_T_2_ (61.03%).

The thermal decomposition process of sample SA_6_T_4_ from the calculated TG curves (Figure 12) was previously described as a three-stage thermal decomposition process: 303.21–428.23 °C for the first stage, 465.74–573.35 °C for the second stage, and 628.40–658.43 °C for the third stage. However, the experimental thermal decomposition behavior of sample SA_6_T_4_ exhibits only a major weight loss peak in the range of 258.63–373.21 °C. The discrepancy between the experimental and calculated TG curves indicates a strong synergistic interaction between APP and talcum [10]. Notably, the thermal decomposition stage corresponding primarily to APP decomposition (465.74–573.35 °C, as observed in the calculated TG curves) is not detected in the experimental curves. This is because the thermal decomposition products of APP have participated in the ceramization reaction. Meanwhile, the presence of APP and talcum also promotes the decomposition of the silicone rubber matrix. Collectively, these results suggest that APP decomposes within the range of 258.63–373.21 °C during the thermal decomposition of sample SA_6_T_4_. The strong synergistic interaction between talcum and the thermal decomposition products of APP alters the pyrolysis behavior of the silicone rubber matrix at high temperatures, thereby increasing the residual yield of sample SA_6_T_4_ and facilitating ceramization.

### 3.8. Ceramization Process and Effects of Ceramifiable Silicone Rubber Composites

**Figure 13:**
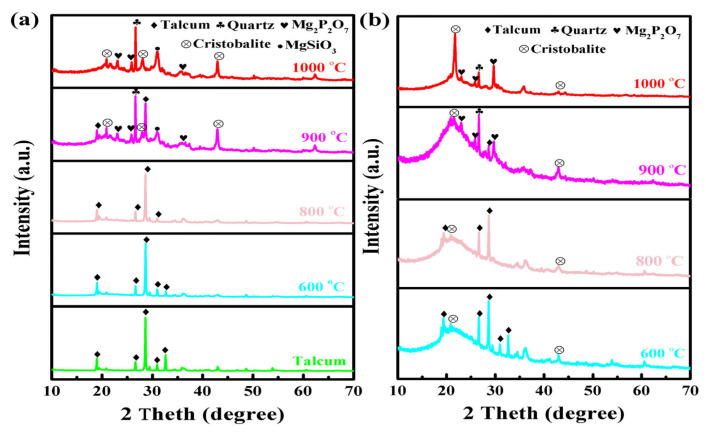
XRD patterns of samples SA_2_T_8_ (**a**) and SA_6_T_4_ (**b**) fired from room temperature to different target temperatures.

XRD analysis was used to illustrate the ceramization process of the ceramifiable silicone rubber composites. Figure 13a shows the XRD results of talcum and the ceramic residues of sample SA_2_T_8_ fired under different conditions. Figure 13b presents the XRD results of the ceramic residues of sample SA_6_T_4_ fired under different conditions. The characteristic diffraction peaks of talcum are observed in its XRD pattern (PDF JCPDS 13-0588). The diffraction peaks of ceramic residues formed at 600 and 800 °C are similar to those of talcum, illustrating that there is primarily the residual talcum phase after ceramification of sample SA_2_T_8_ [22]. However, when sample SA_6_T_4_ was fired at 900 and 1000 °C, a new diffraction peak appeared at 2θ = 31.06°, corresponding to MgSiO_3_, which is attributed to the decomposition of talcum (Equation (2)) [36]. Additionally, new diffraction peaks emerged at 2θ = 23.12, 25.74, and 29.70°, assigned to Mg_2_P_2_O_7_, resulting from the reaction between phosphoric acid and talcum (Equation (3)) [24]. A new peak was detected at 2θ = 26.67°, which is ascribed to the formation of quartz (Equation (4)) [37]. Several weak new peaks appeared at 2θ = 21.87 and 42.96°, corresponding to cristobalite (Equation (4)) [38]. For the residues formed at 1000 °C, the characteristic diffraction peaks of talcum disappeared, indicating the complete melting of talcum. Obviously, the increased content of APP in the silicone rubber/talcum/APP composite played a key role in the ceramization process.

(2) $$Mg_{3}[SiO_{10}](OH_{2})\to MgSiO_{3}+(Si-O-\mathrm{Si})+H_{2}O$$

(3) $$P_{2}O_{5}+MgSiO_{3}\to Mg_{2}P_{2}O_{7}+(Si-O-Si)$$

(4) (Si-O-Si)→Quartz+Cristobalite

**Figure 14:**
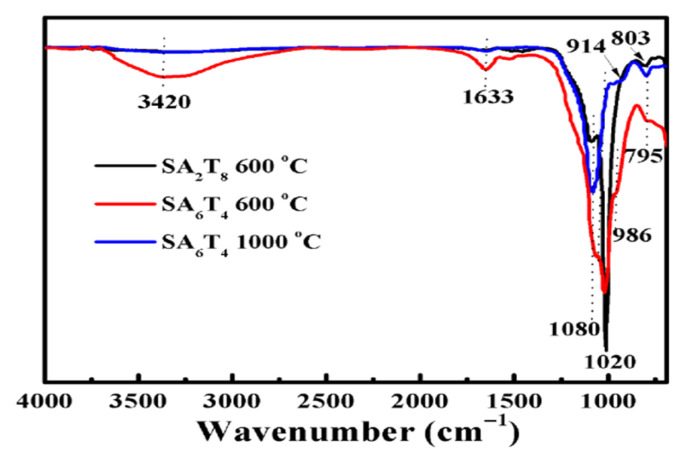
FTIR spectra of the ceramics of sample SA_2_T_8_ and SA_6_T_4_.

The ceramization process was further confirmed through FTIR analysis of the residues formed at different high temperatures, and the results are shown in Figure 14. Peaks at 3420 and 1633 cm^−1^ are assigned to the stretching and bending vibrations of OH groups in P-OH [17,24,39]. The characteristic vibration of P-OH is too weak to be observed in the FTIR spectrum of SA_2_T_8_. For sample SA_6_T_4_, the characteristic vibration of P-OH gradually weakened with increasing temperature, implying that the ceramization process might have undergone in this case. Meanwhile, with the decrease in the characteristic vibration intensity of P-OH, two new peaks appeared at 914 and 986 cm^−1^. These peaks at 914 and 986 cm^−1^ are attributed to P-O-P and PO_3_^−^ groups, respectively. P-O-P and PO_3_^−^ are derived from P_2_O_7_^4−^ [23]. Absorbance peaks corresponding to amorphous silica are observed at 803 cm^−1^ [24,40]. Compared with the FTIR spectrum of sample SA_2_T_8_, a new peak appeared at 795 cm^−1^, whose intensity gradually increased with increasing ceramization temperature, indicating the formation and growth of cristobalite [38]. For both samples SA_2_T_8_ and SA_6_T_4_ fired at 600 °C, two peaks are observed at 1020 and 1080 cm^−1^. However, for sample SA_6_T_4_ fired at 1000 °C, the peak at 1020 cm^−1^ disappeared, implying that the bonding mode between Si and O has changed.

**Figure 15:**
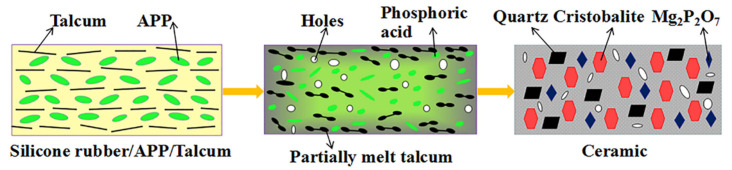
Ceramization process of the ceramifiable silicone rubber composites.

Based on XRF, SEM, TGA, XRD, and FTIR analyses, the possible ceramization mechanism of the ceramifiable silicone rubber composite is revealed as follows. As shown in Figure 15, APP first decomposes to form a viscous melt, in which a large amount of phosphoric acid is generated and talcum is partially melted during thermal decomposition. With increasing temperature, the formed melt bonds talcum and the pyrolysis residues of silicone rubber together via its fluxing effect, accompanied by a crystallization reaction. Finally, a ceramic matrix composed of quartz, cristobalite, and Mg_2_P_2_O_7_ crystals is formed through complex thermal reactions, leading to enhanced high-temperature resistance and ceramifiable properties of the composite.

## 4. Conclusions

A novel ceramifiable silicone rubber composite was successfully fabricated using talcum and eco-friendly ammonium polyphosphate (APP). The composite exhibits excellent electrical insulation. It achieves a UL-94 flame retardant rating of V-0 with a limiting oxygen index (LOI) of 29.4% and self-extinguishes immediately after ignition source removal. The improvement mechanisms for electrical insulation and flame retardancy are mainly ascribed to the talcum platelets, which can disrupt the conductive paths formed by APP and strengthen the condensed-phase barrier effect to inhibit the permeation of heat, oxygen, and flammable gaseous products through a “synergistic effect”. Meanwhile, as an effective synergist, talcum plays an important role in inducing crystallization reactions with APP, thereby improving the high-temperature performance of the ceramic residues including self-supporting property, dimensional stability, and mechanical properties. With superior electrical insulation, flame retardancy and high-temperature ceramization, the composite holds great promise for engineering applications, including new energy battery encapsulation, rail transit sealing/insulation, and wires/cables in harsh environments, ensuring reliable signal transmission and fire safety at extreme temperatures. To the best of our knowledge, this is the first report to synchronously investigate the effects of the talcum and ceramization on the flame retardancy and high-temperature resistance of polymer composites. In summary, this work provides a simple and efficient strategy for improving the flame retardant efficacy of APP, which is conducive to fabricating high-performance polymer composites with simultaneous flame retardancy and high-temperature resistance.

## Figures and Tables

**Table 1:** Formulations of ceramifiable silicone rubber composites (g).

Samples	SR	APP	Talcum	BIPB
Sr	100	-	-	1.5
SA_2_T_8_	100	20	80	1.5
SA_4_T_6_	100	40	60	1.5
SA_6_T_4_	100	60	40	1.5
SA_8_T_2_	100	80	20	1.5

**Table 2:** Volume and surface resistivity of all samples.

Composition	Volume Resistivity (Ω m)	Surface Resistivity (Ω)
Sr	2.51 × 10^16^	2.28 × 10^14^
SA_2_T_8_	2.17 × 10^14^	1.80 × 10^12^
SA_4_T_6_	1.37 × 10^14^	2.82 × 10^12^
SA_6_T_4_	1.38 × 10^13^	4.49 × 10^12^
SA_8_T_2_	2.71 × 10^10^	6.36 × 10^9^

**Table 3:** Flame-retardant properties of all samples.

Composition	LOI (%)	UL-94
Sr	20.5	NR
SA_2_T_8_	24.5	NR
SA_4_T_6_	27.7	V0
SA_6_T_4_	29.4	V0
SA_8_T_2_	29.9	V0

**Table 4:** CC data of all silicone rubber composites at a flux of 50 kW·m^−2^.

Composition	PHRR (kW m^−2^)	THR (MJ m^−2^)	PSPR (1 × 10^−1^ m^2^s^−1^)	Residue (%)
SA_2_T_8_	275.3	24.3	1.8	67.8
SA_4_T_6_	293.3	28.9	1.5	62.3
SA_6_T_4_	250.2	25.9	0.4	65.5
SA_8_T_2_	348.6	27.8	1.0	62.4

**Table 5:** Flexural strength, linear shrinkage, and bulk density of ceramic residues of all ceramifiable silicone rubber composites fired at 900 °C.

Residues	Flexural Strength (MPa)	Linear Shrinkage (%)	Bulk Density (g cm^−3^)
SA_2_T_8_	2.45 ± 0.04	0.66 ± 0.05	0.83 ± 0.02
SA_4_T_6_	2.52 ± 0.02	0.20 ± 0.03	0.98 ± 0.01
SA_6_T_4_	4.81 ± 0.07	−3.75 ± 0.04	1.09 ± 0.04
SA_8_T_2_	2.49 ± 0.05	2.64 ± 0.03	0.53 ± 0.01

## Data Availability

The original contributions presented in this study are included in the article. Further inquiries can be directed to the corresponding author.

## Appendix A

Talcum powders exhibited a lamellar structure, as shown in Figure A1a. There were agglomerations in the talcum powders. APP exhibited a lumpy morphology with irregular boundaries, as shown in Figure A1b.

**Table A1:** Chemical composition of talcum.

Components	SiO_2_	MgO	CaO	Others	LOI ^a^
wt%	46.65	30.21	4.30	0.67	18.17

^a^ The loss on ignition of talcum.

Figure A2 indicates the presence of Si, Mg, and O elements in the selected section. Combined with the chemical composition of talcum (as shown in Table A1), this confirms that the flaky material is talcum. Figure A2 further shows that the talcum is perpendicular to the longitudinal section of the sample and distributes horizontally on the sample surface. This confirms that the talcum has been oriented.
